# Paraneoplastic Neurological Syndromes in Ovarian Cancer: Case Report and Narrative Review for Diagnostic and Clinical Implications

**DOI:** 10.3390/healthcare14131943

**Published:** 2026-07-01

**Authors:** Stefano Restaino, Benedetta Gomba, Yulia Golitsyna, Claudia Andreetta, Elena Poletto, Maria Orsaria, Angelica Tulisso, Giuseppe Scibilia, Giorgio Bogani, Violante Di Donato, Carlo Ronsini, Guglielmo Stabile, Susanna Nicole, Martina Arcieri, Laura Mariuzzi, Lorenza Driul, Maria Rosaria Valente, Giuseppe Vizzielli

**Affiliations:** 1 Clinic of Obstetrics and Gynecology, “Santa Maria Della Misericordia” University Hospital, Azienda Sanitaria Universitaria Friuli Centrale, 33100 Udine, Italy; stefano.restaino@asufc.sanita.fvg.it (S.R.);; 2 PhD School in Biomedical Sciences, Gender Medicine, Child and Women Health, University of Sassari, 07100 Sassari, Italy; 3 Department of Medicine (DMED), University of Udine, 33100 Udine, Italy; gomba.benedetta@spes.uniud.it (B.G.);; 4 Department of Medical Oncology, “Santa Maria Della Misericordia” University Hospital, Azienda Sanitaria Universitaria Friuli Centrale, 33100 Udine, Italy; 5 Department of Medicine, Institute of Pathology, University of Udine, 33100 Udine, Italy; 6 Medicine and Surgery, Kore University, 94100 Enna, Italy; 7 Gynaecological Oncology Unit, Fondazione IRCCS Istituto Nazionale dei Tumori di Milano, 20133 Milano, Italy; 8 Policlinico Umberto I, Department of Maternal and Child Health and Urological Sciences, Sapienza University of Rome, 00161 Rome, Italy; 9 Unit of Gynecologic Oncology, National Cancer Institute, IRCCS, Fondazione “G. Pascale”, 80131 Naples, Italy; 10 Department of Medical and Surgical Sciences, Institute of Obstetrics and Gynecology, University of Foggia, 71121 Foggia, Italy; 11 Clinical Neurology, Azienda Sanitaria Universitaria Friuli Centrale, Presidio Ospedaliero Santa Maria Della Misericordia, 33100 Udine, Italy

**Keywords:** paraneoplastic syndrome, ovarian cancer, onconeural antibodies, encephalitis

## Abstract

**Background**: Paraneoplastic neurological syndromes (PNSs) are rare immune-mediated disorders associated with malignancies and may precede the diagnosis of the underlying tumor. Ovarian cancers, including both epithelial tumors and teratomas, have been associated with a spectrum of antibody-mediated neurological syndromes, although their clinical implications remain poorly defined. **Objective**: To describe three cases of PNS associated with ovarian malignancies and to provide a narrative review of the literature focusing on their diagnostic and clinical implications. **Methods**: We report three patients managed at our institution who developed paraneoplastic neurological syndromes in association with ovarian cancer. In parallel, a narrative review of the literature was conducted through PubMed and Google Scholar to identify studies published between 2020 and 2025 reporting PNS in ovarian malignancies. Relevant studies were selected and analyzed qualitatively, with emphasis on timing of onset, clinical presentation, antibody profiles, treatment strategies, and outcomes. **Results**: Eighteen studies were included, the majority of which were case reports. In most cases, neurological symptoms preceded the diagnosis of ovarian cancer, highlighting their potential role as an early clinical indicator of malignancy. Cerebellar degeneration was the most frequent presentation, often associated with anti-Yo antibodies, while encephalitis was more commonly linked to anti-NMDAR antibodies. Our clinical cases illustrate the heterogeneity of presentation and the complexity of diagnosis and management, requiring a multidisciplinary approach. Across studies, outcomes were variable, with partial improvement or stabilization observed in most cases following oncologic and immunomodulatory treatment. **Conclusions**: Paraneoplastic neurological syndromes may represent an early clinical clue to ovarian malignancy. Increased awareness among clinicians is essential to prompt appropriate diagnostic evaluation and timely oncologic management. Further research is needed to clarify the role of onconeural antibodies in early detection and disease monitoring.

## 1. Introduction

Paraneoplastic neurological syndromes (PNSs) are rare immune-mediated disorders associated with malignancies and not directly related to tumor invasion, metastasis, or treatment-related toxicity. They are thought to result from an autoimmune response triggered by tumor-associated antigens that cross-react with neuronal tissues, leading to neurological dysfunction [1,2].

The clinical spectrum of PNS is highly heterogeneous and may involve both the central and peripheral nervous systems, manifesting as cerebellar degeneration, limbic encephalitis, sensory neuropathies, and other neurological syndromes [1,2]. Among gynecological malignancies, ovarian tumors have been associated with several PNSs, most notably anti-N-methyl-D-aspartate receptor (anti-NMDAR) encephalitis in teratomas and anti-Yo-associated cerebellar degeneration in epithelial ovarian cancer [3,4,5].

A key clinical aspect of PNSs is that neurological symptoms may precede the diagnosis of the underlying malignancy. In such cases, PNSs may represent the first clinical manifestation of cancer, prompting diagnostic investigations that ultimately lead to tumor detection [6]. This highlights their potential role as an early clinical indicator of malignancy.

Despite increasing recognition, the clinical implications of PNS in ovarian cancer remain poorly defined. Most available evidence is limited to case reports and small case series, and the role of onconeural antibodies in diagnosis, surveillance, and prognostication is still unclear [5,7].

In this context, we report three cases of ovarian malignancies associated with paraneoplastic neurological syndromes and provide a narrative review of the literature, with the aim of highlighting their clinical presentation, diagnostic challenges, and potential implications for patient management.

## 2. Materials and Methods

### 2.1. Study Design

This study consists of two components: (1) a descriptive analysis of three clinical cases of paraneoplastic neurological syndromes associated with ovarian malignancies, and (2) a narrative review of the literature aimed at summarizing current evidence on the clinical presentation, diagnostic work-up, and management of these conditions.

### 2.2. Case Reports

We retrospectively identified three patients diagnosed with paraneoplastic neurological syndromes in association with ovarian malignancies and managed at the Gynecological Department of Santa Maria della Misericordia Hospital, Udine, Italy. The aim is to provide real-world clinical evidence supporting existing knowledge and to highlight the importance of multidisciplinary management.

Clinical data were collected from electronic medical records and included demographic characteristics, oncologic history, neurological presentation, imaging findings, laboratory and cerebrospinal fluid analyses, treatment strategies, and follow-up.

Data collection was performed in accordance with local institutional policies and applicable regulations. This study was conducted according to institutional ethical standards. Institutional Review Board approval was obtained (IRB: 42/2026), and written informed consent for publication of clinical data was obtained whenever feasible.

### 2.3. Narrative Literature Review

A narrative review of the literature was conducted to summarize current evidence on paraneoplastic neurological syndromes associated with ovarian malignancies, with particular focus on clinical presentation, diagnostic timing, antibody profiles, therapeutic strategies, and outcomes.

A literature search was performed in PubMed and Google Scholar to identify relevant studies published between January 2020 and March 2025. The following search terms were used: (“ovarian cancer” OR “ovarian neoplasm”) AND (“paraneoplastic neurological syndromes” OR “paraneoplastic neurologic syndrome” OR “PNS”). No geographical restrictions were applied.

Titles and abstracts were screened for relevance, followed by full-text assessment of potentially eligible articles. Studies were considered for inclusion if they reported clinical or observational data on patients with ovarian malignancies presenting with paraneoplastic neurological syndromes.

### 2.4. Study Selection

This narrative review was conducted using a systematic approach based on critical selection to improve transparency in study identification and inclusion. Titles and abstracts were independently screened for relevance, followed by full-text assessment of potentially eligible studies.

Studies were included if they:

- Reported clinical or observational data on patients with ovarian malignancies and associated PNSs.
- Provided information on at least one of the following: clinical presentation, diagnostic work-up, antibody profile, treatment, or outcomes.
- Eligible study designs included case reports, case series, retrospective studies, and clinically relevant reviews providing information on neurological manifestations, antibody profiles, diagnostic work-up, treatment strategies, or outcomes.

Studies were excluded if they:

- Focused on non-gynecological malignancies;
- Described paraneoplastic syndromes without neurological involvement;
- Reported neurological conditions that are not clearly associated with ovarian malignancy;
- Did not provide clinically relevant information for the purposes of this review.

The article selection process is clarified through a flow chart diagram in the Supplementary Materials.

### 2.5. Data Extraction and Synthesis

From each included study, the following variables were extracted: year of publication, study design, sample size, patient age (when available), timing of paraneoplastic neurological syndrome onset in relation to ovarian cancer diagnosis, diagnostic methods, antibody profile, clinical presentation, treatment strategies, and outcomes.

Data extraction was performed in a standardized manner, and findings were synthesized qualitatively. Given the rarity of the condition and the heterogeneity of the available evidence, no quantitative synthesis or meta-analysis was performed.

Results were organized and reported according to key thematic domains, including timing of onset, clinical manifestations, diagnostic tools, antibody profiles, therapeutic strategies, and outcomes.

## 3. Case Report 1

A 60-year-old woman with a previous diagnosis of breast cancer in 2008 was referred to our Department in March 2024 after receiving a histological diagnosis of advanced-stage high-grade serous carcinoma in pelvic lymph node sampling. She underwent cytoreductive surgery with definitive diagnosis of high-grade serous ovarian cancer with pelvic and abdominal lymph node metastasis (FIGO stage III). Slightly before surgery, the patient started complaining of peripheral dysesthesia, firstly associated with previous breast tumor management. Platinum-based chemotherapy followed primary surgery, with an initial favorable response. During oncological therapies, the patient developed subacute neurological deterioration characterized by progressive cerebellar dysfunction and worsening balance disturbances. Neurological examination revealed truncal ataxia, dysmetria, and impaired coordination. Brain magnetic resonance imaging (MRI) was performed, with no evidence of metastatic disease or acute structural abnormalities. Given the clinical suspicion of a paraneoplastic neurological syndrome, a comprehensive diagnostic evaluation was initiated, including cerebrospinal fluid analysis and blood tests. The level of CA125 was >100 U/mL. Cerebrospinal fluid (CSF) analysis demonstrated inflammatory changes without evidence of infection or malignant cells. Serum testing revealed the presence of anti-Yo antibodies, supporting the diagnosis of paraneoplastic cerebellar degeneration.

A multidisciplinary team involving neurologists, gynecologic oncologists, and immunologists was engaged to guide management. The patient received immunomodulatory therapy, including corticosteroids (prednisone) and intravenous immunoglobulins, alongside oncologic surveillance to exclude tumor recurrence.

Despite treatment, neurological symptoms only partially improved, with persistent cerebellar impairment affecting the patient’s functional autonomy. However, early recognition of the syndrome allowed stabilization of the neurological condition and facilitated coordinated multidisciplinary care.

This case highlights the importance of considering PNS in patients with unexplained cerebellar symptoms, even in the absence of radiological evidence of disease.

## 4. Case Report 2

A 58-year-old woman presented with rapidly progressive neurological symptoms, including diplopia, vertigo, and worsening coordination.

Neurological evaluation revealed signs of cerebellar dysfunction and ocular motor abnormalities. Initial neuroimaging did not demonstrate metastatic involvement of the central nervous system. However, due to the rapid progression of neurological deficits, a paraneoplastic neurological syndrome was suspected. Laboratory investigations included serum and CSF analysis for onconeural antibodies. Testing revealed positivity for anti-Yo antibodies, confirming the diagnosis of paraneoplastic cerebellar degeneration. PET-CT was performed with evidence of captant inguinal lymph nodes, so the patient underwent guided lymph node biopsy. Immunohistochemistry on needle biopsy tissue revealed a gynecological cancer profile.

The patient underwent comprehensive oncologic staging (MRI and whole-body CT scan), which confirmed the presence of ovarian serous carcinoma, without evidence of distant metastases. Considering low PFS and autonomous degeneration, the patient was referred to the oncologic department and underwent neoadjuvant chemotherapy for 3 cycles (carbotaxole). Surgical cytoreduction was then performed, with a chemotherapy response 3, followed by adjuvant systemic therapy in accordance with current oncologic standards. The final stage of the disease, according to FIGO staging, was IIIa.

Concomitantly, the patient received immunomodulant therapy administered with prednisone to control the immune process. Although partial neurological improvement was observed following treatment, residual cerebellar deficits persisted during follow-up.

These findings highlight the diagnostic complexity of paraneoplastic neurological syndromes and underscore the importance of early recognition and prompt multidisciplinary management.

This case underscores the potential role of PNS as an early manifestation of ovarian malignancy and the importance of prompt multidisciplinary evaluation.

## 5. Case Report 3

A 27-year-old patient with no previous medical history came to our attention for abdominal–pelvic pain. Gynecological examination revealed a left ovarian solid mass wider than 10cm, extending to the uterus and involving the right adnexa. She underwent surgery with left annessiectomy and peritoneal biopsies. Histological examination revealed a high-grade immature ovarian teratoma. In a few days, she presented to primary care because of a sudden epileptic seizure. Encephalic CT excluded vascular or ventricular anomalies, and MRI with gadolinium revealed normal parenchymal representation without neuronal damage. The results of cultural and bacterial analyses of hematic samples were completely negative, while both CSF and blood samples analysis revealed high levels of anti-NMDAR antibody. EEG showed diffuse encephalitic activity. Neurologists administered 1 g of methylprednisolone for 5 days and levetiracetam as symptomatic treatment; the patient was referred to our gynecological department for surgical completion. Restaging surgery revealed peritoneal disease localization. The multidisciplinary strategy aimed to prioritize immunoglobulin treatment due to neurological worsening, so the patient was discharged a month after from the neurological department, with improved conditions, and maintenance therapy with rituximab was prescribed. Due to neurological improvement, chemotherapy treatment with cisplatin, etoposide and bleomycin was administered for 4 cycles in order to reduce peritoneal disease extension. A post-treatment CT scan revealed persistence of disease; neurological status is currently improved but not regressed. Common agreement is to continue neurological and gynecological follow-up and to perform a CT scan every 6 months. The patient is alive with the disease and under strict control.

## 6. Literature Review

During preliminary research, 64 studies were selected. A total of 18 studies were included in the analysis, including 17 single-case reports and one retrospective study, reflecting the rarity of paraneoplastic neurological syndromes (PNSs) in ovarian malignancies [1,8] (Table 1).

### 6.1. Timing of Onset

Across the available literature, PNSs frequently preceded the diagnosis of ovarian cancer [26,27]. Among studies reporting this information, neurological symptoms occurred before tumor detection in most cases, whereas a smaller proportion developed during or after oncologic treatment [8,28,29,30]. These findings suggest that PNSs may represent an early clinical manifestation of ovarian malignancy and should prompt immediate oncologic evaluation.

### 6.2. Clinical Manifestations

Cerebellar syndromes emerged as the most common clinical presentation, typically manifesting as ataxia, dysarthria, and balance impairment, and most frequently associated with anti-Yo antibodies [1,9,15,30,31,32]. Encephalitic presentations, including psychiatric symptoms and seizures, were the second-most-common pattern and were often linked to anti-NMDAR antibodies [13,14,33]. Less frequent manifestations included opsoclonus-myoclonus syndrome and, rarely, stroke-like presentations [18,34]. Overall, cerebellar involvement appears to represent the predominant clinical pattern in ovarian cancer-associated PNSs.

### 6.3. Diagnostic Work-Up

Magnetic resonance imaging was the most commonly used diagnostic tool, although it was frequently non-specific or normal, particularly in early stages [12]. Computed tomography was mainly employed to exclude alternative diagnoses such as vascular events or brain metastases, while cerebrospinal fluid analysis and antibody testing provided supportive but not definitive diagnostic information [2,7,9,14]. These findings highlight the limited sensitivity of imaging and the central role of clinical suspicion in establishing the diagnosis.

### 6.4. Antibody Profile

Although anti-Yo antibodies were the most frequently reported, a wide range of antibody profiles was observed, including anti-NMDAR, anti-Hu, anti-Zic, anti-CV2, and anti-AMPAR antibodies [1,9,14,15,23]. Notably, a substantial proportion of cases showed no detectable antibodies [9,13]. This variability reflects the heterogeneity of the underlying immune response and limits the diagnostic specificity of antibody testing.

### 6.5. Therapeutic Strategies

Management typically involves a combination of oncologic treatment and immunomodulatory therapy. Corticosteroids and intravenous immunoglobulins were the most commonly used approaches, sometimes combined with monoclonal antibodies such as rituximab [1,9,10,13,14,15,16]. Surgical treatment of the underlying tumor was frequently associated with stabilization or improvement of neurological symptoms [24,30,33]. Overall, early initiation of combined oncologic and immunomodulatory treatment appears to be associated with better neurological outcomes. Sometimes, immunoglobulin treatment was initiated during neoadjuvant chemotherapy, although no improvement was obtained [25].

### 6.6. Outcomes

Clinical outcomes were heterogeneous. Partial improvement or stabilization was the most common result, whereas complete neurological recovery was relatively rare [1,9,15]. Mortality was reported in a minority of cases, particularly in anti-Yo-associated cerebellar degeneration [19,35]. Overall, neurological outcomes remain suboptimal, highlighting the severity of these syndromes [22,24,25].

## 7. Discussion

This study provides a focused overview of paraneoplastic neurological syndromes (PNSs) associated with ovarian malignancies, combining three illustrative clinical cases with a narrative synthesis of the recent literature. Our findings confirm the heterogeneous nature of PNSs and reinforce their clinical relevance as potential early indicators of underlying malignancy [1,2,30]. As clarified in PNS reclassification published by Graus F. et al. in 2021 [2], anti-Yo is considered a high-risk antibody (cancer relation frequency >70%), while anti-NMDAR is an intermediate-risk antibody (cancer relation frequency of 38%); this suggests that neurological syndromes involving these molecules are related to ovarian cancer, respectively, in more than 70% and 38% of cases.

A key observation emerging from both our cases and the reviewed literature is that neurological symptoms frequently precede the diagnosis of ovarian cancer. This temporal relationship highlights the importance of considering PNSs as a diagnostic “red flag,” particularly in patients presenting with unexplained cerebellar or neuropsychiatric symptoms. Notably, when these symptoms lead to the identification of the underlying malignancy, the ovarian cancer is often already at an advanced stage, highlighting the diagnostic challenges associated with this disease and the importance of maintaining a high index of suspicion in patients presenting with paraneoplastic neurological syndromes. Early recognition may prompt targeted oncologic investigations, potentially leading to earlier diagnosis and treatment [8,28,29,30].

From a mechanistic perspective, PNS can be broadly classified according to the nature of the target antigen. Syndromes associated with intracellular antigens, such as anti-Yo, are primarily mediated by cytotoxic T-cell responses leading to irreversible neuronal damage, which may explain the limited response to immunotherapy and the generally poor neurological recovery observed in these patients. In contrast, syndromes associated with neuronal surface antibodies, such as anti-NMDAR, are predominantly antibody-mediated and tend to show a more favorable response to immunomodulatory treatment. This distinction is clinically relevant, as it may influence both therapeutic decisions and prognosis.

In terms of clinical presentation, cerebellar syndromes appear to be the most common manifestation, often associated with anti-Yo antibodies, while encephalitic presentations are more frequently linked to anti-NMDAR antibodies [1,9,15,30,33]. This pattern reflects the underlying immune-mediated pathophysiology and suggests a degree of correlation between antibody profile and neurological phenotype. However, the variability in antibody detection observed across studies underscores the complexity of these syndromes and limits their use as standalone diagnostic tools [2,9,13,14,15].

**Figure 1:**
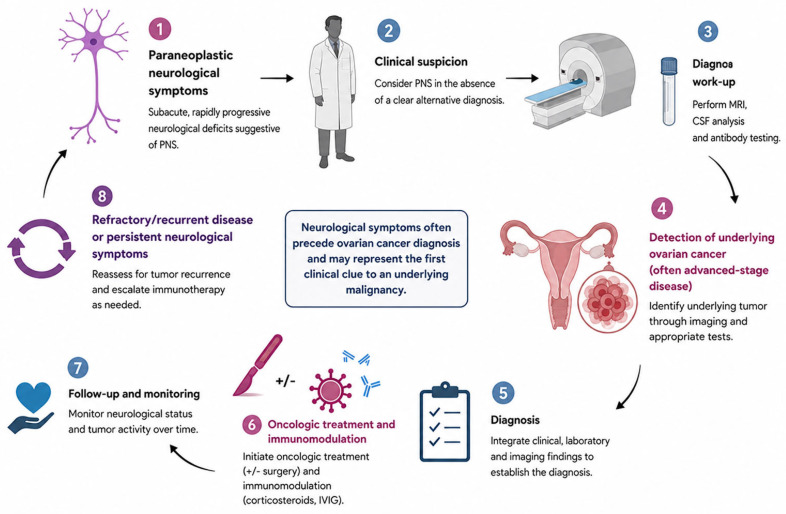
Proposed clinical timeline of paraneoplastic neurological syndromes in ovarian malignancies. Neurological symptoms may precede tumor diagnosis and act as an early clinical clue prompting oncologic evaluation. Early multidisciplinary management is essential to optimize outcomes.

The diagnostic work-up of PNS remains challenging. Neuroimaging, particularly magnetic resonance imaging, is frequently non-specific or even normal, while cerebrospinal fluid analysis and antibody testing can support the diagnosis but are not always conclusive [2,7,9,12,14]. In this context, clinical suspicion remains central, and a multidisciplinary approach involving neurologists, oncologists, and gynecologists is essential [1,29,33]; Figure 1 resumes possible diagnostic workup in case of suspicion. PNS diagnostic criteria should always be considered [2] in order to distinguish paraneoplastic neurological disease from other neurological complications arising from oncological treatments.

Therapeutically, our data reinforce the importance of a combined approach targeting both the tumor and the immune response. Corticosteroids and intravenous immunoglobulins represent the most commonly employed strategies, with variable outcomes [1,9,10,13,14,15,16,23]. These treatments are intended to suppress or modulate the immune response elicited by the tumor, thereby limiting the autoimmune mechanisms responsible for neuronal injury and the development of neurological symptoms. Although some patients experience stabilization or partial improvement, complete neurological recovery is uncommon, particularly in anti-Yo-associated syndromes, which are often characterized by irreversible neuronal damage [9,15,19,31,35].

Our findings also highlight the potential role of PNSs as markers of disease activity. In some cases, the onset or progression of neurological symptoms may reflect tumor presence or recurrence, suggesting a possible role for paraneoplastic antibodies in disease surveillance. However, current evidence is insufficient to support their routine use as biomarkers, and further studies are needed [1,30].

**Figure 2:**
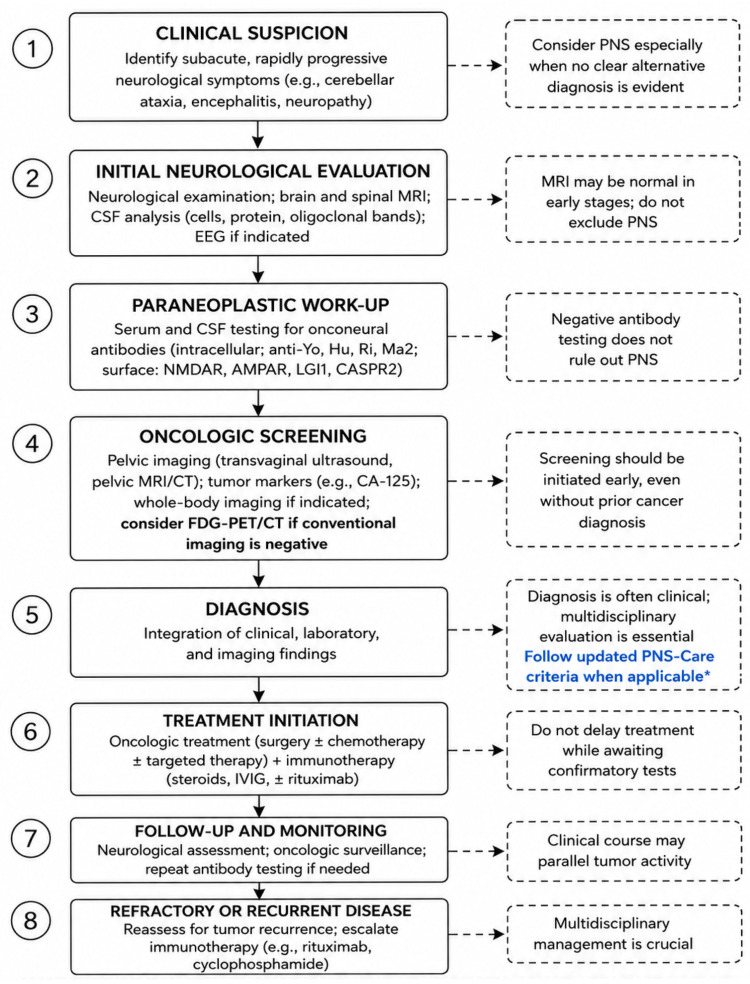
Detailed flow chart of proposed diagnostic work-up in patients presenting with neurological symptoms suggesting PNSs. * PNS Care Criteria: [2].

Based on our findings and the available literature, a pragmatic clinical pathway can be proposed. In patients presenting with rapidly progressive cerebellar or encephalitic syndromes, particularly when standard diagnostic work-up is unrevealing, clinicians should promptly consider a paraneoplastic etiology. First-line evaluation should include brain MRI, cerebrospinal fluid analysis, and comprehensive onconeural antibody testing. However, given the limited sensitivity of these investigations, a negative result should not exclude the diagnosis. In parallel, early oncologic screening—such as pelvic imaging in women—should be initiated to identify a potential underlying malignancy. Treatment should not be delayed pending confirmatory tests, especially in cases with high clinical suspicion. A hypothetical diagnostic work-up is reported in Figure 2.

Despite these insights, several limitations should be acknowledged. First, the available evidence is largely based on case reports and small series, limiting the strength of conclusions. Second, the heterogeneity of clinical presentations, antibody profiles, and treatment strategies precludes quantitative synthesis. Finally, the retrospective nature of our case series and the narrative design of this review introduce potential selection and reporting bias.

In conclusion, paraneoplastic neurological syndromes represent a rare but clinically significant manifestation of ovarian malignancies. Their recognition is crucial, as they may precede tumor diagnosis and significantly impact patient management. Increased awareness and a multidisciplinary approach are essential to optimize diagnostic and therapeutic strategies. Future research should focus on better defining the role of paraneoplastic antibodies in early detection and disease monitoring.

### 7.1. In the Context of Other Malignancies

Paraneoplastic neurological syndromes are associated with malignancies other than ovarian cancer and are most commonly linked to small-cell lung carcinoma, breast carcinoma, thymoma, testicular tumors, and lymphomas [20,36]. Clinical manifestations may involve the central nervous system (including limbic and extra-limbic encephalitis, encephalomyelitis, brainstem syndromes, and rapidly progressive cerebellar ataxia), the peripheral nervous system (such as subacute sensory neuronopathy and polyradiculoneuropathy), the neuromuscular junction (e.g., Lambert–Eaton myasthenic syndrome), and muscle (e.g., dermatomyositis). Notable associations include anti-Hu (ANNA-1) antibodies with small-cell lung carcinoma, anti-Ri with breast cancer, anti-Ma2 with testicular neoplasms, and CRMP5 antibodies with thymoma or lung cancer [37]. These disorders generally present with a subacute and rapidly progressive course and may precede the diagnosis of the underlying malignancy. Early recognition and prompt treatment are essential for improving outcomes, although prognosis remains variable depending on the specific antibody profile and associated neoplasm [36].

### 7.2. Clinical Implications

Paraneoplastic neurological syndromes should be considered in the differential diagnosis of patients presenting with unexplained neurological symptoms, particularly cerebellar or neuropsychiatric manifestations. In this context, clinicians should maintain a high index of suspicion for an underlying malignancy, including ovarian cancer, even in patients without a prior oncologic history.

The findings of this study support the importance of early multidisciplinary evaluation, involving neurologists, gynecologic oncologists, and immunologists, to facilitate timely diagnosis and management. Prompt recognition of PNSs may allow earlier identification of occult malignancy and initiation of appropriate oncologic and immunomodulatory treatment.

Although cerebrospinal fluid analysis and onconeural antibody testing can support the diagnosis, treatment should not be delayed in clinically suspected cases, given the potential for rapid neurological deterioration and irreversible damage.

### 7.3. Future Perspectives

Paraneoplastic neurological syndromes in ovarian malignancies remain poorly understood, and current evidence is limited by small sample sizes and heterogeneous study designs. Future research should aim to establish standardized diagnostic criteria and management algorithms to improve clinical outcomes.

Particular attention should be given to the potential role of onconeural antibodies as biomarkers for early cancer detection and disease monitoring. Prospective studies are needed to clarify their diagnostic and prognostic value, as well as their potential integration into clinical practice.

In addition, advances in immunology and molecular oncology may provide further insights into the pathophysiology of PNSs and identify novel therapeutic targets.

## 8. Conclusions

Paraneoplastic neurological syndromes represent a rare but clinically relevant manifestation of ovarian malignancies. They may precede tumor diagnosis and, therefore, serve as an important clinical clue to underlying cancer.

Recognition of these syndromes is essential to enable timely diagnostic evaluation and appropriate multidisciplinary management. Greater awareness among clinicians may contribute to earlier diagnosis and improved patient care. Further studies are required to better define the role of paraneoplastic antibodies in early detection and disease monitoring, and to optimize diagnostic and therapeutic strategies in this complex clinical setting.

## Figures and Tables

**Table 1:** Literature review: data extraction.

Authors	Year/Type	N Patients, Age	PNS Before/After OC	Main Diagnostic Tool	Antibody	Symptoms	Therapeutic Strategy	Outcome
Ray M.D. et al. [1]	2025 retrospective study	19 patients, 55 yo	/	TC-MRI	Anti-Yo, anti-Hu, and anti-NMDAR	Cerebellar degeneration	Symptomatic; corticosteroids	Improved if complete response
Rashid S. et al. [9]	2025 case report	1 patient, 73 yo	After	TC-MRI-CSF analysis	anti-Hu; anti-Zic	Ataxia; dysarthria	Corticosteroid	Partial response
Szczupak M. et al. [10]	2025 case report	1 patient, 20 yo	Before	MRI-CT-EEG-gyn ultrasound	None	Opsoclonus-myoclonus	IVIg, corticosteroids, and antiepileptics	Resolution
Ye T. et al. [11]	2025 case report	1 patient, 70 yo	Before	CT-CSF	Anti-Yo	Cerebellar degeneration	Neoplasm surgery	Improvement
Tierney M. et al. [12]	2024 case report	1 patient, 58 yo	Before	MRI	/	Cerebellar degeneration	Symptomatic	Stabilization of symptoms
Sudhakaran S. et al. [13]	2024 case report	2 patients, 50–23	Before	MRI-CSF analysis	Anti-Yo; anti-NMDAR	Cerebellar degeneration/psychiatric	IVIg; corticosteroids	Persistent symptoms/lost to follow-up
Huang Y. et al. [14]	2023 case report	1 patient, 53 yo	After	MRI-CSF analysis	Anti-AMPAR	Limbic dysfunction	IVIg, corticosteroids, and rituximab	Improvement
Giucca A. et al. [15]	2023 case report	1 patient, 60 yo	After	MRI-TC-CSF analysis	Anti-Yo	Cerebellar degeneration	IVIg and corticosteroids	Death
Jones A. et al. [16]	2022 case report	1 patient, 27	After	MRI-CSF analysis-PET	None	Opsoclonus-myoclonus	IVIg, corticosteroids, and rituximab	Complete resolution
Berlingieri D. et al. [17]	2022 case report	1 patient, 13 yo	Before	EEG-TC-MRI-CSF	Anti-NMDAR	Encephalitis	IVIg, corticosteroids, and surgery	Improvement
Tandi R. et al. [18]	2022 case report	1 patient, 76 yo	Before	MRI-TC	None	Stroke	Thrombolysis and pelvic surgery	Improvement
Greenlee J. et al. [19]	2022 commentary	1 patient	Before	CT, autopsy	Anti-Yo	Cerebellar degeneration	None	Died before surgery
Kim et al. [20]	2023 retrospective study	94 + 203	/	TC-MRI-CSF	Anti-NMDAR	Epileptic seizures	Surgery	No neurological improvement
Cecchi E. et al. [21]	2020 case report	1 patient, 50 yo	After	Analysis	Anti-Yo	Cerebellar degeneration	IVIg; plasma exchange	Improvement
Liapi A. et al. [22]	2020 case report	1 patient, 61 yo	Before	CSF analysis	Anti-Yo	Cerebellar degeneration	CHT; surgery	No neurological improvement
Juárez-Vignon Whaley JJ et al. [23]	2021 case report	1 patient, 62 yo	After	PET-TC-CSF analysis	Anti-CV2	Cerebellar degeneration	Corticosteroids	/
Wang X. et al. [24]	2025 case report	1 patient, 65 yo	Before	TC-CSF analysis	Anti-Yo	Cerebellar degeneration	Surgery, IVIg, and steroids	No neurological improvement
Apama K. et al. [25]	2024 case report	1 patient, 68 yo	Before	CSF-TC	Anti-Yo	Cerebellar degeneration	CHT + IVIg then surgery	No neurological improvement

## Data Availability

The data presented in this study are available upon request from the corresponding author due to privacy and ethical policy.

## Supplementary Materials

The following supporting information can be downloaded at https://www.mdpi.com/article/10.3390/healthcare14131943/s1, PRISMA Flow Diagram.
