# Supplementary figures and images for: Paraneoplastic Neurological Syndromes in Ovarian Cancer: Case Report and Narrative Review for Diagnostic and Clinical Implications
Source: Healthcare (Basel). 2026 Jul 1;14(13):1943. doi: 10.3390/healthcare14131943 (PMC13362078; doi:10.3390/healthcare14131943)

PRISMA Flow Diagram

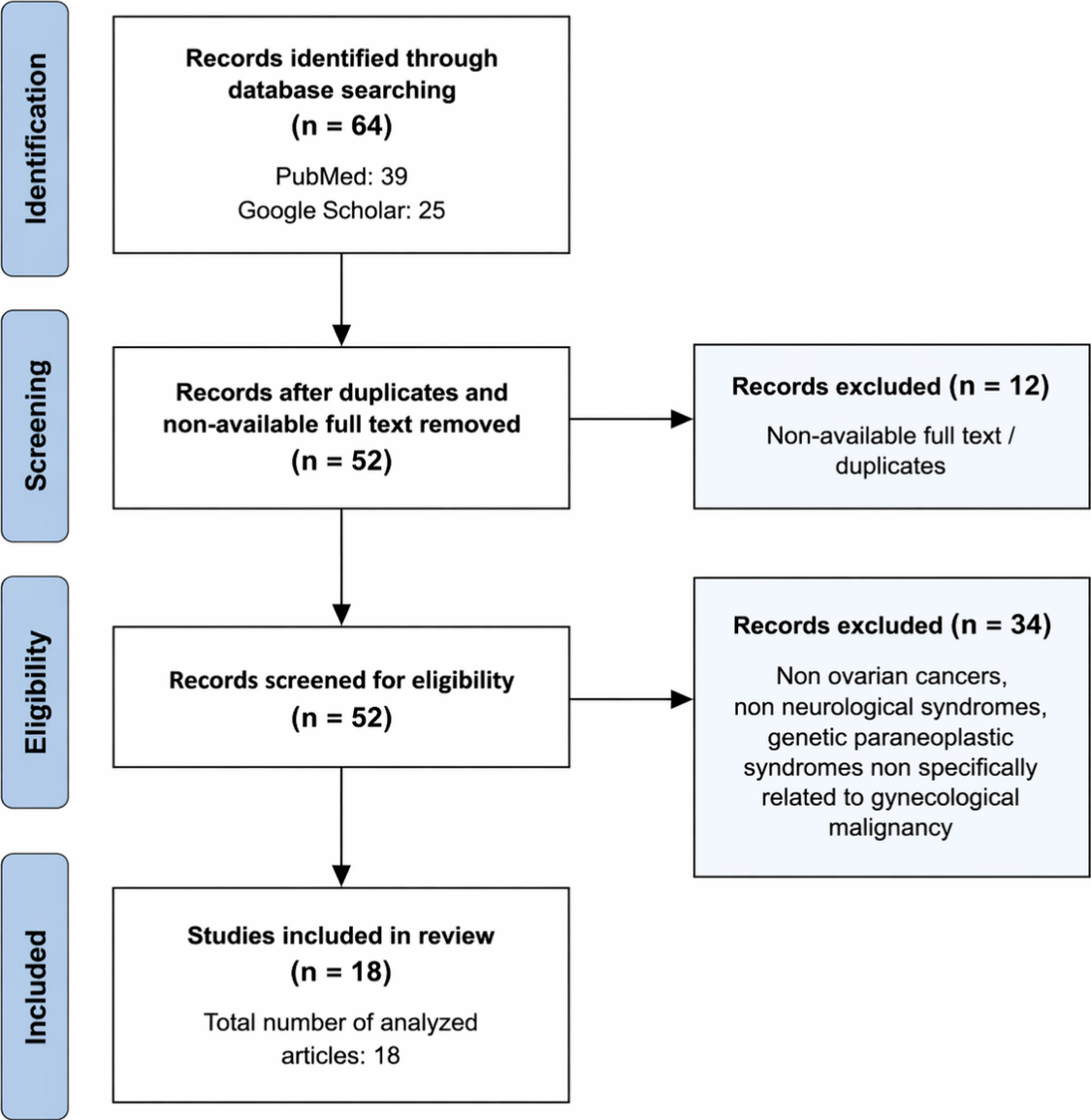

Supplement: Supplementary file 1 [file healthcare-14-01943-s001.zip › healthcare-4295280-supplementary.pdf]
